# Interplay between different forms of power and meritocratic considerations shapes fairness perceptions

**DOI:** 10.1038/s41598-022-15613-9

**Published:** 2022-07-06

**Authors:** Giannis Lois, Arno Riedl

**Affiliations:** 1 Department of Microeconomics and Public Economics, School of Business and Economics, Maastricht University, 6200 MD Maastricht, The Netherlands; 2 Maastricht University—Center of Neuroeconomics, Maastricht, The Netherlands; 3 CESifo, IZA, and Maastricht University, Maastricht, The Netherlands

**Keywords:** Human behaviour, Psychology

## Abstract

Power imbalance often leads to unequal allocations. However, it remains largely unknown how different forms of power and meritocratic considerations interact to shape fairness perceptions. Using modified Ultimatum Games, we examined how two power forms—decision power and availability of attractive outside option—affect bargaining behavior and fairness perceptions, and how meritocratic considerations are incorporated into the fairness perceptions of powerful and powerless individuals. We identified an asymmetric power effect: having increased decision power or attractive outside options independently increased self-advantageous allocations and self-serving fairness perceptions, whereas the combined lack of both power forms led to self-disadvantageous allocations but had no influence on fairness perceptions. The power effect on fairness perceptions became symmetric when power was obtained through a meritocratic process (procedural justice). In contrast, relative contributions to resource production (distributive justice) did not moderate power effects. We provide causal evidence that the powerful, but not the powerless, strive to minimize cognitive dissonance between behavior and fairness perceptions by interpreting fairness in self-serving ways. This study contributes novel insights into the interplay between different power forms, the asymmetry of power effects, the moderating role of procedural justice, and the mediating role of behavior in the power-driven adjustment of fairness perceptions.

## Introduction

Power, defined as one’s ability to pursue one’s preferred outcome^1^ permeates most social interactions. When bargaining over resources, imbalance of power may result in economic inequalities^2,3^. Given that people care about fairness, an emerging and relatively unaddressed question is how the interplay between different forms of power and meritocratic considerations influence fairness perceptions of unequal allocations. In light of growing economic inequality worldwide^4^, this question is extremely relevant as the perceived fairness of unequal outcomes determines—to a large extent—the public demand for wealth and income redistribution^5^.

In bargaining situations, power is often conceptualized in two different forms: decision power, in the sense of having more agency in the bargaining process, or power due to the availability of attractive outside options in case of negotiation breakdown^1^. Previous studies have examined the independent effects of these two forms of power^6–9^ but have ignored their potential interplay. A prominent example of the interplay between these two power forms is wage bargaining^10^. The decline of labor unions and the emerging gig economy have undermined employees’ ability to affect wages^11,12^. At the same time, in countries with high unemployment rate and no safety net, employees’ powerless position relative to employers is further compromised by the absence of attractive outside options. The combined presence or absence of these two power forms may exert synergistic effects that influence not only employers’ and employees’ bargaining behavior but also their perceptions of fair wages. The present study constitutes the first experimental attempt to investigate the interplay between these two power forms on behavior and fairness perceptions in a dyadic bargaining setting.

In everyday bargaining situations most people find themselves in powerless positions^13^. However, previous experimental work has mainly focused on powerful individuals who tend to make self-advantageous allocations and form self-serving fairness perceptions^6–9,14^. The emphasis on the powerful reflects the dominant assumption that power and the lack thereof represent opposite ends of the same continuum^15^. This assumption has led to methodological limitations that impede the systematic investigation of asymmetric power effects^16^. One important concern is the absence of appropriate control conditions that would allow drawing inferences about the effects of powerlessness. Even the few studies that focused on powerlessness report inconsistent results. Some studies found that powerless individuals tolerate exploitation from the powerful^17^, form self-undermining fairness perceptions^9^, and legitimize economic and social inequalities when they perceive themselves as powerless^18^. However, other evidence is consistent with an asymmetric power effect in that the powerful behave selfishly and adopt self-serving fairness perceptions, whereas the powerless opt for relatively equal allocations of resources which they deem as fair^8,19,20^. In light of the limitations and discrepancies of this literature, this study systematically compares the behavior and fairness perceptions of powerful and powerless individuals.

Apart from power, fairness perceptions are also shaped by meritocratic considerations. People care about the fairness of an allocation outcome (i.e., distributive justice)^21–23^ and the fairness of the process that determines an allocation outcome (i.e., procedural justice)^24–27^. Field and experimental work on distributive justice has shown that people switch from a strict egalitarian principle to an equity principle (distributions based on effort and contributions) when the latter can justify a self-advantageous allocation^21–23,28^. This opportunistic switch between fairness ideals is often accomplished by the self-serving incorporation of information about merit (i.e., personal effort and contributions). Specifically, people exploit advantageous information, discount disadvantageous information, or self-servingly interpret ambiguous information about merit to justify selfish actions^29–31^. Given that powerful individuals often behave more selfishly than powerless individuals, they are more likely to exploit advantageous merit information in self-serving ways to justify their behavior. This assumption alludes to a potential interaction between power and meritocratic considerations that to date remains unexplored. We aim to fill this gap by examining how powerful and powerless individuals incorporate advantageous, disadvantageous, and ambiguous contribution information into their bargaining behavior and their fairness perceptions.

In the presence of power imbalance, meritocratic considerations may also pertain to the procedures that determine the assignment of power positions (i.e., procedural justice)^24^. Previous studies have shown that when power imbalance reflects effort rather than luck, people are more likely to accept low status positions^25,26^ and less likely to support redistribution of resources^27,32^. This study aims to extend this line of work by investigating whether powerful and powerless individuals are equally sensitive to procedural justice when bargaining over resources and when evaluating the fairness of bargaining outcomes.

A large body of evidence suggests that people allocate resources by trading off their material self-interest with their desire to perceive themselves as fair^22^. According to this view, cognitive dissonance motivates people to minimize the distance between their behavior and their fairness perceptions, often by interpreting fairness in self-serving ways^33^. This dissonance mechanism fits well with evidence that powerful individuals adopt self-serving fairness perceptions to justify their selfish behavior^9^. However, it is unclear whether this mechanism also motivates powerless individuals to form self-undermining fairness perceptions (“*I deserve less”)* in order to justify self-disadvantageous allocations that they are often forced to tolerate. Such a self-undermining adjustment of fairness perceptions would conflict with the ever-present self-enhancement bias^34^. In light of these two counteracting processes, this study explores whether behavior mediates the power-driven adjustment of fairness perceptions for both powerful and powerless individuals.

Taken together, the aim of the present study is threefold. First, we aim to provide a multidimensional investigation of power effects on fairness perceptions by focusing on the interplay between two common forms of power and the potential asymmetry of power effects. Second, we conceptualize merit on the basis of both distributive and procedural justice and we examine its moderating role in the relationship between power and fairness perceptions. Third, we delve deeper into the underlying mechanism of the power-driven adjustment of fairness perceptions by examining the role of cognitive dissonance in this process.

The framework for accomplishing the aforementioned aims involves modified Ultimatum Games (UGs) with joint-production^22,35^. Our experimental setup cleanly separates decision power (i.e., proposer vs responder) from power derived from attractive outside options. In three experiments, we parametrically manipulate the size of the disagreement payoffs (DPs) which are the payoffs that materialize when bargaining between the proposer and the responder breaks down. This operationalization of power gives players with attractive outside options (i.e., large DP) the opportunity to leave a social interaction when the proposed bargaining outcome is not satisfactory^1^. We also implement an “equal power” control condition, where the DPs of proposers and responders are equal. This control condition allows the systematic investigation of asymmetric power effects.

Based on existing evidence on power effects^6–9^, we hypothesize that the relatively powerful (i.e., having large DP or being a proposer) would opt for more self-advantageous allocations and form self-serving fairness perceptions (Hypothesis 1). In contrast, the relatively powerless (i.e., having small DP or being a responder) would tolerate self-disadvantageous allocations (Hypothesis 2a). Importantly, whether the powerless would also form self-undermining fairness perceptions is an open question, because two plausible counteracting processes (i.e., cognitive dissonance vs self-enhancement bias) may pull them into opposite directions. Cognitive dissonance between behavior and beliefs may lead to a self-undermining adjustment of fairness perception to justify the tolerated self-disadvantageous allocations^9^, while a self-enhancement bias^34^ may impede this process (competing hypotheses 2b).

In Experiment 1, power positions due to outside options are either determined by luck or by participants’ effort in a real-effort task (i.e., procedural justice). In line with previous work on power legitimacy^25–27^, we hypothesize that procedural justice would provide justifications for a self-serving exploitation of power imbalances which will amplify the power effects on bargaining behavior and fairness perceptions (Hypothesis 3). In Experiment 2, we operationalize merit as participants’ relative contribution to resource production (i.e., distributive justice). We provide participants advantageous, disadvantageous, or ambiguous information about their relative contribution to resource production. Given the well-documented tendency to exploit ambiguous or advantageous information^29–31^, we hypothesize that people, especially those in powerful positions would predominantly respond to advantageous contribution information by increasing their self-serving fairness perceptions (Hypothesis 4a). In contrast, powerless individuals may try to minimize dissonance between bargaining behavior and fairness perceptions and thus respond predominantly to disadvantageous information by reporting more self-undermining fairness perceptions (Hypothesis 4b). We also explore whether ambiguous information is incorporated differently depending on the power position.

Lastly, according to the dissonance hypothesis^33^, bargaining behavior would mediate the effects of the two power forms (i.e., size of DP or bargaining role) on fairness perceptions (Hypothesis 5). In Experiment 3, we seek to establish the causal mediating role of bargaining behavior by introducing a control condition whereby the computer plays the UG on behalf of the participants. The computer condition nullifies the experience of dissonance between bargaining behavior and fairness perceptions and thus eliminates any behavior-mediated power effects on fairness perceptions (Hypothesis 6).

## Methods

We conducted three experiments through the online platform Academic Prolific. All participants were at least 18 years old, UK residents, and had high approval rate in this platform (i.e., above 97% completion rate). Participants were paid a fixed participation fee (5£ per hour) and received their UG earnings as bonus payment. In all experiments, a priori power analysis was carried out to determine the sample size that would provide us with 80% power to detect medium effect sizes *η*_*p*_^2^ = 0.06 for main effects and interaction effects at the *α* = 0.05 after correcting for multiple comparisons. The study was approved by the Ethical Review Committee Inner City Faculties of Maastricht University (Reference No. ERCIC_215_30_09_2020_A). All experiments were performed in accordance with the relevant guidelines and regulations of Maastricht University for studies with human participants. Before conducting the experiments, we obtained informed consent from all participants. The hypotheses, sampling procedure, analyses, and exclusion criteria for all three experiments were preregistered on OSF (Experiment 1: https://osf.io/s5yt3, Experiment 2: https://osf.io/y5tzn, Experiment 3: https://osf.io/7wfy8). Additional information about participants’ exclusion, procedures, and detailed tables of participants’ behavior and fairness perceptions are reported in the “Supplementary Material”.

### Experiment 1

The a priori power analysis suggested a target sample of 240 participants (20 per condition). We recruited 315 participants but due to several incomplete observations, our final sample size was *n* = 247 (149 women, Age: *M* = 38.35 years, *SD* = 11.70). Participants played a one-shot UG as proposers or responders with joint surplus production. First, they performed a Counting Zeros Task^36^ to produce a surplus of 100 Experimental Currency Units (ECUs). This real-effort task consisted of counting, within two minutes, the number of zeros that are contained in twelve 5 × 5 tables of numbers. Both the proposer and the responder had to successfully complete this task to produce the surplus of 100 ECUs. Following the successful surplus production, proposers and responders bargained over the produced surplus. We implemented the strategy method^37^ in which proposers offer an amount from 0 to 100 ECUs to responders and responders indicate the minimum offer they are willing to accept without knowing the actual offer the proposers made. This operationalization has been widely used in experiments^38^ and eliminates anchoring effects on observed proposals and provides responders the same choice space as proposers.

Unlike the standard UG, in this modified UG, the DPs of proposers and responders differed from zero. More specifically, proposers and responders were randomly assigned to one of three DP conditions (between-subjects design). In the large-DP and small-DP conditions, powerful participants were informed that their DP was 60 ECUs, whereas their powerless partners were informed that their DP was 10 ECUs. In the equal-DP condition, both the proposer and the responder had a DP of 35 ECUs. Following the UG, proposers and responders reported their fairness perceptions by indicating the allocation they perceive as fairest under their respective DP conditions.

In this experiment, we operationalized merit as the legitimacy of their power positions (i.e., procedural justice). More specifically, participants were either assigned to the different DP conditions based on their performance in a real-effort task that they performed in a previous session (effort condition), or this assignment was random (luck condition). Details about the assignment to DP conditions can be found in the “Supplementary Material”.

We conducted a three-way factorial ANOVA with the size of DPs (large-DP, equal-DP, and small-DP), bargaining role (proposer and responder), and merit (luck and effort) as between-subject independent variables. Participants’ bargaining behavior and their fairness perceptions were used as dependent variables. Proposers’ bargaining behavior was measured as the amount they keep for themselves (i.e., Amount Kept for Self or AKS). Responders’ bargaining behavior was measured as the minimum offer that they are willing to accept from proposers (i.e., Minimal Acceptable Offer or MAO). Fairness perceptions were measured as the number of ECUs that proposers or responders find fair to keep for themselves. We also conducted simple mediation analyses to test whether bargaining behavior mediates the relationship between the two forms of power (i.e., size of DP and bargaining role) and fairness perceptions. We used the equal-DP condition as a reference to examine the effects of having large and small DP.

### Experiment 2

The a priori power analysis suggested a target sample of 360 participants (20 per condition). We recruited 426 participants but due to several incomplete observations our final sample size was *n* = 372 (283 women, Age: *M* = 33.97 years, *SD* = 10.77). The procedure was similar to Experiment 1 with some modifications. In the surplus-production task, participants were instructed to count the zeros in as many tables as possible within two minutes. To produce the surplus of 100 ECUs, the number of correctly counted tables by both proposer and responder needed to exceed a certain threshold. The threshold was unknown to the participants and thus both the proposer and the responder needed to perform to the fullest of their capacity to increase the chances of producing the surplus.

In Experiment 2, we manipulated merit by providing participants information about their and their partner’s contribution to surplus production (i.e., distributive justice). Participants in the advantageous (disadvantageous) information condition received truthful information that they contributed more (less) than their UG partner. In the ambiguous information condition, we informed participants that one of the UG players (either themselves or their UG partner) contributed more to the surplus production without revealing who. After receiving this information, participants bargained over the produced surplus (UG), and then stated their fairness perceptions as in Experiment 1.

We performed a three-way factorial ANOVA using the size of DPs, bargaining roles, and merit (advantageous, ambiguous, and disadvantageous information) as between-subject independent variables. Similar to Experiment 1, bargaining behavior and fairness perceptions were used as dependent variables. We conducted simple mediation analyses to test whether bargaining behavior mediates the power effects (effects of size of DP or bargaining role) on fairness perceptions. We used the equal-DP condition as a reference to examine both effects of having large and small DP.

### Experiment 3

In Experiment 3, we used the design of the luck condition of Experiment 1 (referred here as human condition), but we eliminated participants’ agency in the UG by introducing a control condition where the computer played the UG on behalf of the participants. The a priori power analysis suggested a target sample of 120 participants (20 per condition). We recruited 142 participants but due to incomplete observations, we achieved a final sample size of *n* = 123. The total sample (human condition of Experiment 1 and computer condition of this experiment) was *n* = 247 (162 women, Age: *M* = 36.46 years, *SD* = 11.15).

The computer condition differed from the human condition in that proposers and responders were informed that the computer will make an offer or set a MAO, respectively, on behalf of them. For each DP condition and for each bargaining role, computer’s bargaining behavior was matched with the actual bargaining behavior of proposers and responders in the human condition. This design eliminates any anchoring-related explanations since participants in both the computer and human condition faced the same bargaining behavior before reporting their fairness perceptions.

We conducted a moderated mediation analysis to test whether bargaining behavior mediates the power effects (effects of size of DP and bargaining role) on fairness perceptions and whether participants’ agency moderates the relationship between bargaining behavior and fairness perceptions (Model 14)^39^. We used the equal-DP condition as a reference to examine both effects of having large and small DP.

## Results

### Experiment 1

Experiment 1 employed a factorial design to examine the effects of the size of DP, bargaining role and merit (i.e., procedural justice) on bargaining behavior and fairness perceptions. The three-way ANOVA on bargaining behavior revealed that increasing the size of DPs leads to higher AKS and higher MAOs (*F*(2, 235) = 53.78, *p* < 0.001, *η*_*p*_^*2*^ = 0.31, 95% CI [0.22, 0.40]) and that proposers’ AKS are, on average, higher than responders’ MAOs (*F*(1, 235) = 70.71, *p* < 0.001, *η*_*p*_^*2*^ = 0.23, 95% CI [0.14, 0.32]). The omnibus test also yielded a significant two-way interaction of the two power forms on bargaining behavior (*F*(2, 235) = 4.67, *p* = 0.010, *η*_*p*_^*2*^ = 0.04, 95% CI [0.00, 0.09]). The DP effect on proposers’ AKS was significant (*F*(2, 116) = 24.04, *p* < 0.001, *η*_*p*_^*2*^ = 0.29, 95% CI [0.16, 0.41]) but asymmetric. Proposers with large DP kept significantly more ECUs for themselves than equal-DP proposers, whereas the AKS of small-DP proposers and equal-DP proposers did not differ (Table 1). On the other hand, large DP and small-DP responders set MAOs which are significantly higher and lower than equal-DP responders, respectively (*F*(2, 119) = 32.63, *p* < 0.001, *η*_*p*_^*2*^ = 0.35, 95% CI [0.22, 0.46]) (Table 1). Procedural justice did not affect bargaining behavior across all power conditions (no main or interaction effect).

**Table 1:** Comparison of bargaining behavior across power conditions.

Role	Comparison between DP conditions		Mean difference in bargaining behavior (ECUs)	95% confidence interval for difference	
				Lower bound	Upper bound
Proposers’ offers	Equal DP	Large DP	− 8.516***	− 12.824	− 4.207
		Small DP	3.835	− 0.529	8.199
Responders’ MAOs	Equal DP	Large DP	− 6.205*	− 11.852	− 0.559
		Small DP	12.728***	7.010	18.445

*p < 0.05.

**p < 0.01.

***p < 0.001.

**Figure 1:**
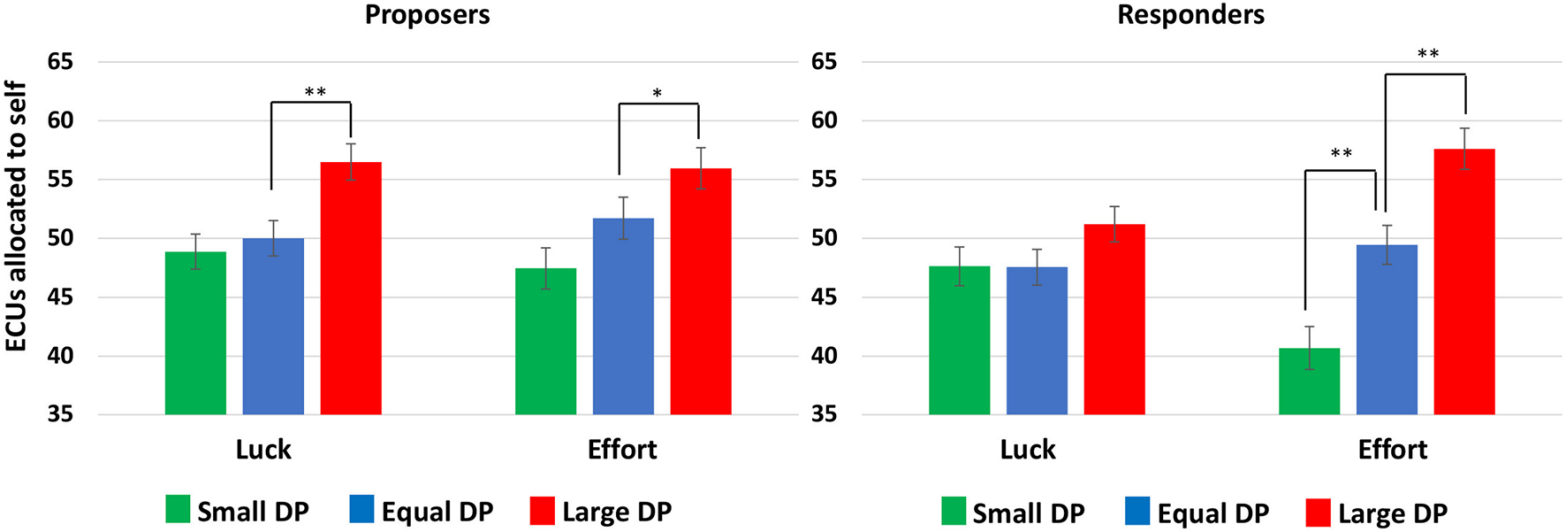
Share of self-allocated surplus (in ECUs) that is considered fair across power and merit conditions. Error bars represent standard error of the mean. Significant differences between equal-DP and large-DP or small-DP conditions are represented by asterisks: **p < 0.01, *p < 0.05.

The three-way ANOVA on fairness perceptions showed that increasing the size of DP leads to more self-serving fairness perceptions (*F*(2, 35) = 31.10, *p* < 0.001, *η*_*p*_^*2*^ = 0.21, 95% CI [0.12, 0.29]) and that proposers’ fairness perceptions are more self-serving than those of responders (*F*(1, 235) = 8.22, *p* = 0.005, *η*_*p*_^*2*^ = 0.03, 95% CI [0.00, 0.09]). Furthermore, we observed a significant three-way interaction effect between the two power forms and merit (*F*(2, 235) = 3.55, *p* = 0.030, *η*_*p*_^*2*^ = 0.03, 95% CI [0.00, 0.08]). Follow-up analysis indicated a significant two-way interaction between the size of DP and merit in responders (*F*(2, 119) = 7.38, *p* = 0.001, *η*_*p*_^*2*^ = 0.11, 95% CI [0.02, 0.21]) but not in proposers (*F*(2, 116) = 0.56, *p* = 0.538, *η*_*p*_^*2*^ = 0.01, 95% CI [0.00, 0.06]). Planned comparisons revealed an asymmetric DP effect on proposers’ fairness perceptions irrespective of merit (*F*(2, 116) = 14.26, *p* < 0.001, *η*_*p*_^*2*^ = 0.20, 95% CI [0.08, 0.31]). Specifically, the fairness perceptions of equal-DP proposers did not differ from those of small-DP proposers but were less self-serving than those of large-DP proposers (Fig. 1). On the other hand, fairness perceptions of equal-DP responders differed significantly from both large-DP and small-DP responders, albeit only in the merit condition (*F*(2,119) = 22.44, *p* < 0.001, *η*_*p*_^*2*^ = 0.27, 95% CI [0.14, 0.39]) (Fig. 1).

These findings support Hypothesis 1 in that possessing power through large DPs or by being a proposer (vs a responder) independently increased self-advantageous allocations and self-serving fairness perceptions. On the other hand, the combined lack of both power forms (responders with small DPs) led to self-disadvantageous allocations (Hypothesis 2a) but had no impact on proposers’ or responders’ fairness perceptions (Hypothesis 2b). Consistent with Hypothesis 3, procedural justice amplified the power (i.e., size of DP) effects on fairness perceptions but only for responders. This significant three-way interaction was also reflected in the fact that self-undermining fairness perceptions were only reported by responders who were assigned small DPs based on merit (Hypothesis 2b).

**Figure 2:**
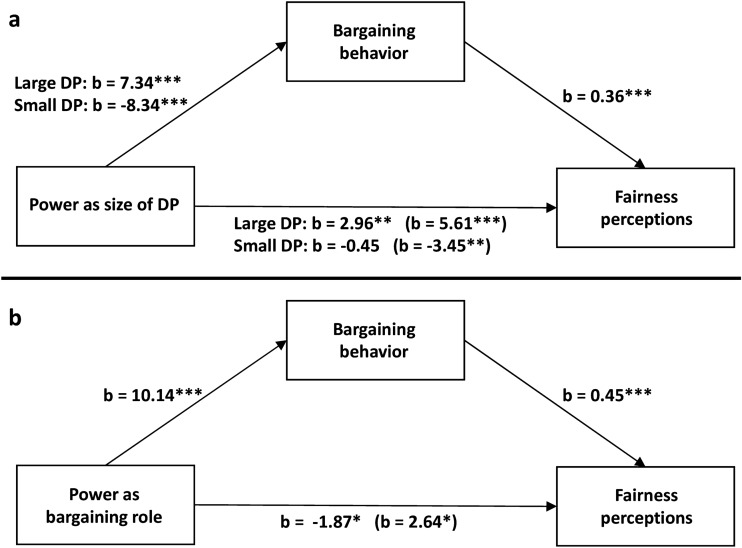
Simple mediation analyses. Regression coefficients for the relationship between the size of DP (**a**) or bargaining role (**b**) and fairness perceptions as mediated by bargaining behavior. The effects of the size of DP and bargaining role on fairness perceptions controlling for bargaining behavior are in parentheses. Significant relationships between variables are represented by asterisks: ***p < 0.001, **p < 0.01, *p < 0.05.

Hypothesis 5 was supported as the simple mediation analysis revealed that bargaining behavior partially mediated the self-serving adjustment of fairness perception which is driven by large DPs (ES = 2.64, 95% CI [1.22–4.59]) and fully mediated the self-undermining adjustment of fairness perceptions which is driven by small DPs (ES = -3.00, 95% CI [− 4.96 to − 1.56]) (Fig. 2a). Bargaining behavior also fully mediated the bargaining role effect on fairness perceptions (ES = 4.51, 95% CI [2.86–6.54]) (Fig. 2b).

### Experiment 2

Experiment 2 employs a similar design as Experiment 1 but instead of procedural justice, contribution to surplus production is used as a benchmark for merit (distributive justice). Consistent with results in Experiment 1, the three-way ANOVA on bargaining behavior revealed that increasing the size of DPs leads to more self-advantageous allocations (*F*(2,354) = 42.58, *p* < 0.001, *η*_*p*_^*2*^ = 0.194, 95% CI [0.123, 0.262]) and that proposers’ AKS is higher than responders’ MAOs (*F*(1,354) = 52.32, *p* < 0.001, *η*_*p*_^*2*^ = 0.129, 95% CI [0.070, 0.194]. The omnibus test also revealed a two-way interaction effect of the two power forms on bargaining behavior (*F*(2,354) = 5.49, *p* = 0.004, *η*_*p*_^*2*^ = 0.030, 95% CI [0.003, 0.070]). Follow up analysis revealed that the DP effect is present in both proposers (*F*(2,181) = 11.28, *p* < 0.001, *η*_*p*_^*2*^ = 0.111, 95% CI [0.035, 0.195]) and responders (*F*(2,173) = 33.66, *p* < 0.001, *η*_*p*_^*2*^ = 0.280, 95% CI [0.169, 0.375]). However, proposers’ AKS was significantly higher in the large-DP condition compared to the equal-DP condition but did not differ significantly between equal-DP and small-DP conditions (Table 2). On the other hand, large-DP (small-DP) responders set higher (lower) MAOs compared to equal-DP responders (Table 2). Moreover, merit influenced bargaining behavior, such that compared to ambiguous information, advantageous (disadvantageous) information about contribution to surplus production increased (decreased) self-advantageous allocations (*F*(2, 354) = 6.34, *p* = 0.002, *η*_*p*_^*2*^ = 0.06, 95% CI [0.00, 0.01]).

**Table 2:** Comparison of bargaining behavior across power conditions.

Role	Comparison between DP conditions		Mean difference in bargaining behavior (ECUs)	95% confidence interval for difference	
				Lower bound	Upper bound
Proposers’ offer	Equal DP	Large DP	− 5.510**	− 9.519	− 1.500
		Small DP	1.972	− 1.960	5.904
Responders’ MAOs	Equal DP	Large DP	− 7.132**	− 11.705	− 2.559
		Small DP	8.045***	3.511	12.579

*p < 0.05.

**p < 0.05.

***p < 0.001.

**Figure 3:**
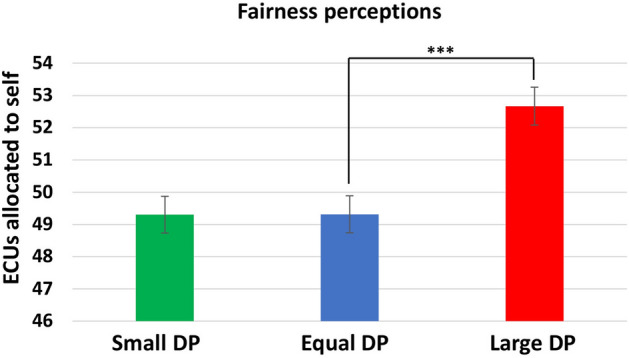
Share of self-allocated surplus (in ECUs) that is considered fair across power conditions. Error bars represent standard error of the mean. Significant difference between equal-DP and large-DP conditions is represented by asterisks: ***p < 0.001.

The three-way ANOVA on fairness perceptions revealed that increasing the size of DPs leads to more self-serving fairness perceptions (*F*(2,354) = 12.87, *p* < 0.001, *η*_*p*_^*2*^ = 0.068, 95% CI [0.024, 0.120]), and that proposers’ fairness perceptions are more self-serving than those of responders (*F*(1,354) = 7.77, *p* = 0.006, *η*_*p*_^*2*^ = 0.021, 95% CI [0.002, 0.060]). Furthermore, compared to ambiguous information, advantageous (disadvantageous) information about contribution to surplus production increased (decreased) self-serving fairness perceptions (*F*(2, 354) = 17.05, *p* < 0.001, *η*_*p*_^*2*^ = 0.09, 95% CI [0.04, 0.15]). All three-way or two-way interactions were non-significant. Planned comparisons showed an asymmetric effect in that fairness perceptions of equal-DP individuals were less self-serving than those of large-DP individuals (ES = 3.36, *p* < 0.001, 95% CI [1.38, 5.33]) but did not differ from those of small-DP individuals (Fig. 3).

Taken together, the DP and bargaining role effects on bargaining behavior and fairness perceptions are consistent with those found in Experiment 1. Being a proposer and having large DPs led to more self-advantageous allocations and self-serving fairness perceptions (Hypothesis 1), whereas small DPs reduced responders’ MAOs (Hypothesis 2a) but had no impact on proposers’ or responders’ fairness perceptions (Hypothesis 2b). However, contrary to Hypothesis 4 and unlike the case of procedural justice in Experiment 1, information about contribution to surplus production (distributive justice) did not moderate the relationship between the two forms of power and fairness perceptions.

**Figure 4:**
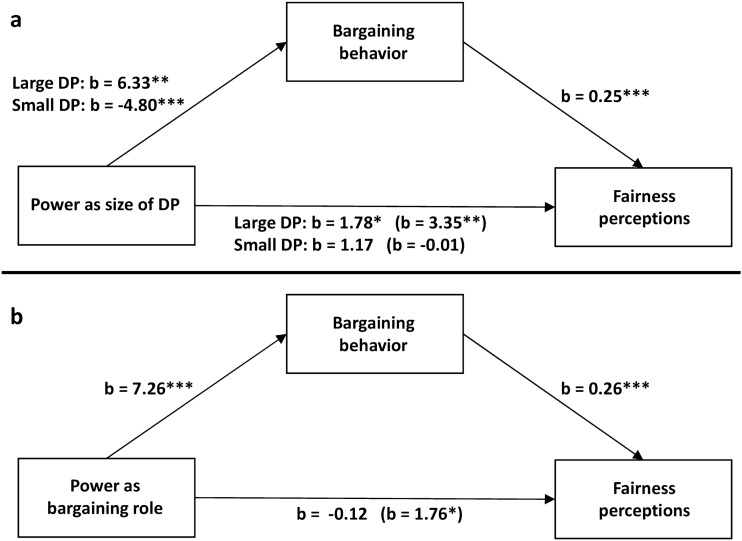
Simple mediation analyses. Regression coefficients for the relationship between the size of DP (**a**) or bargaining role (**b**) and fairness perceptions as mediated by bargaining behavior. The effects of the size of DP and bargaining role on fairness perceptions controlling for bargaining behavior are in parentheses. Significant relationships between variables are represented by asterisks: ***p < 0.001, **p < 0.01, *p < 0.05.

Consistent with Hypothesis 5 and the findings of Experiment 1, the mediation analysis revealed a significant indirect effect of both small DP (ES = − 1.19, 95% CI [− 1.94, − 0.55]) and large DP (ES = 1.57, 95% CI [0.70, 2.73]) on fairness perceptions through bargaining behavior (Fig. 4a). Similarly, bargaining behavior fully mediated the relationship between bargaining role and fairness perceptions (ES = 1.89, 95% CI [1.10–2.84]) (Fig. 4b).

### Experiment 3

**Figure 5:**
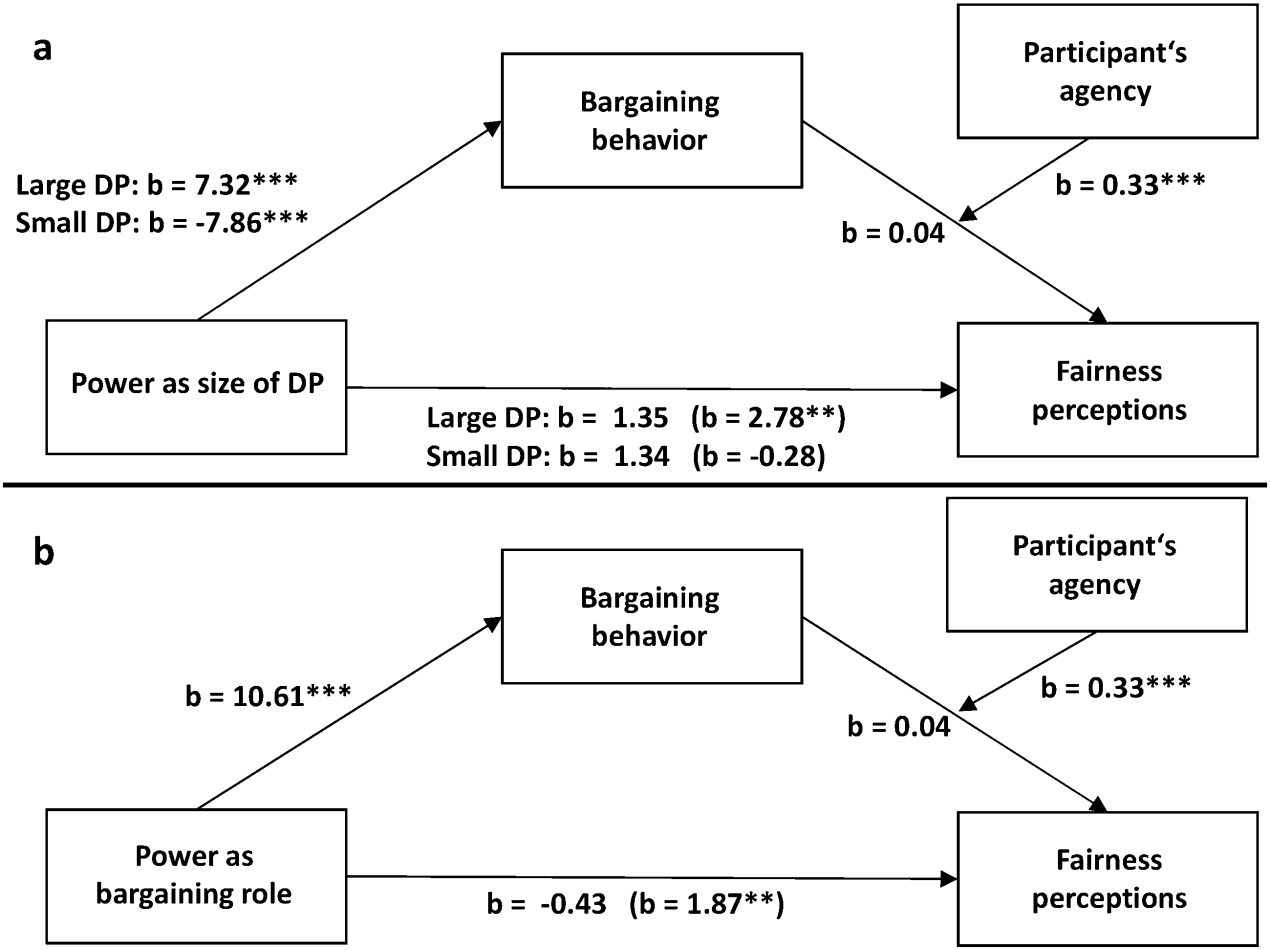
Moderated mediation analyses. Regression coefficients for the relationship between the size of DP (**a**) or bargaining role (**b**) and fairness perceptions as mediated by bargaining behavior and participants’ agency moderating the relationship between bargaining behavior and fairness perceptions. The effects of the size of DP and bargaining role on fairness perceptions controlling for bargaining behavior are in parentheses. Significant relationships between variables are represented by asterisks: ***p < 0.001, **p < 0.01, *p < 0.05.

Experiment 1 and 2 provided correlational evidence for the mediating role of bargaining behavior. Experiment 3 employs a design similar to Experiment 1 but aims to infer the causal role of participants’ behavior in the power-driven adjustment of fairness perceptions by eliminating participants’ agency in a treatment where offers where generated by the computer. The moderated mediation analysis yielded significant indices of moderated mediation for both large-DP (ES = 2.44, 95% CI [0.97, 0.44]) and small-DP (ES = − 2.62, 95% CI [− 4.33, − 1.16]) conditions (Fig. 5a). The conditional indirect effects were significant only in the human condition for both large-DP (ES = 2.73, 95% CI [1.21, 4.73]) and small-DP (ES = − 2.93, 95% CI [− 4.70, − 1.48]) conditions. Similarly, the moderated mediation analysis with bargaining role as independent variable (Fig. 5b) yielded a significant moderated mediation index (ES = 3.53, 95% CI [1.51, 5.77]). The conditional indirect effect of bargaining role was significant only in the human condition (ES = 4.03, 95% CI [2.07, 6.27]). These findings support Hypothesis 6 and suggest that participants’ bargaining behavior plays a causal mediating role in the relationship between the two forms of power (i.e., size of DP or bargaining role) and fairness perceptions.

## Discussion

The present study addresses several gaps in the existing literature on how the interplay between different forms of power and meritocratic considerations influence the distribution of resources and fairness perceptions about these distributions. We investigated two different power forms -decision power due to the bargaining role and power due to the availability of attractive outside options- and found that they independently lead to more self-advantageous allocations and self-serving fairness perceptions. Conversely, people accept self-disadvantageous allocations only when their limited decision power (i.e., responders) is combined with unattractive outside options. Interestingly, even in this extremely powerless position, participants predominantly deemed equal allocations as fair. Only when outside options were determined through a meritocratic process (procedural justice), responders, but not proposers, with unattractive outside options adopted self-undermining interpretations of fairness. On the other hand, information about contributions to surplus production (distributive justice), did not moderate the power effects on bargaining behavior and fairness perceptions. Importantly, we also established the causal mediating role of bargaining behavior in the power-driven adjustment of fairness perceptions.

These findings are consistent with previous work showing that these two forms of power exert independent effects on bargaining behavior^6,14,40^ and fairness perceptions^7–9,41^. By directly comparing these two power forms we conclude that both power forms are good predictors of bargaining behavior but the availability of attractive outside options (i.e., DP) is a stronger predictor of fairness perceptions (i.e., effect sizes are three to seven times larger). However, this conclusion should be taken with caution as other manipulations of decision power and availability of outside options may yield different results. Moreover, self-disadvantageous allocations are only accepted by individuals who have both limited decision power and unattractive outside options. The synergism between these forms of power suggests that the mere focus on decision power (i.e., bargaining roles) and inattention to available outside options does not capture the full picture of the forces determining bargaining outcomes in many important bargaining situations (e.g., wage negotiations) where different forms of power or the lack thereof coexist^10^.

Furthermore, these results indicate an asymmetric power effect on fairness perceptions. Even extremely powerless individuals (i.e., lacking both forms of power) did not perceive disadvantageous inequality as fair, whereas powerful individuals consistently adopted self-serving interpretations of fairness. In line with this pattern, a previous study found that fairness perceptions differed more between a full-power and equal-power condition than an equal-power and no-power condition^8^. A plausible explanation for this asymmetry may be the ever-present self-enhancement bias that hinders the formation of self-undermining beliefs and facilitates the endorsement of self-serving beliefs^34^. This explanation is consistent with studies showing that people are more averse to disadvantageous compared to advantageous inequality^42^ and they process positive and negative violations of the equality principle in different ways^43,44^. It is worth noting that other experimental studies found that relatively powerless individuals justify unfair outcomes^9^ and abusive behavior^17^. However, both these studies lack appropriate equal-power control conditions that would allow the systematic investigation of power asymmetries. In this respect, our study demonstrates a robust asymmetry in power effects which is present irrespective of other, orthogonal forms of power individuals may possess.

Our results also highlight the role of procedural justice in amplifying the power effects on fairness perceptions. There is empirical evidence that people deem equality of opportunity as important when judging the fairness of allocations^24,45^. At a larger scale, the perceived legitimacy of established power hierarchies enhances the justification of social and economic inequalities^18^. Consistent with this literature, our findings show that procedural justice affects fairness perceptions but not necessarily bargaining behavior. Another experiment where bargaining roles were either assigned based on luck or earned through effort also reported no procedural justice effects on behavior^46^. These results raise the question of whether the legitimacy of both power forms (bargaining roles and attractiveness of outside options) selectively alters fairness perceptions without affecting bargaining behavior. In our setting, only responders’ fairness perceptions were sensitive to procedural justice, whereas proposers treated legitimate and illegitimate power forms indistinguishably in their fairness perceptions. One reason for this selective sensitivity is that possessing decision power (even when it is due to luck) may shift one’s attention to this form of power neglecting other power forms and their legitimacy. This idea is supported by evidence that the powerful (in this case proposers) ignore the unfairness of procedures that lead to self-advantageous outcomes^40,47^ and pay attention to information that justifies their position^48^. A potential implication of this finding is that extremely powerless individuals, who lack both legitimate and illegitimate forms of power, are more likely to justify power abuse and accept inequalities that result from illegitimate power hierarchies. This preliminary conclusion warrants further investigation of the potential role of procedural justice in justifying the outcomes of not only legitimate but also illegitimate power hierarchies.

Unlike procedural justice, information pertaining to distributive justice had no moderating role on power effects. Advantageous and disadvantageous contribution information equally affected participants’ bargaining behavior and fairness perceptions irrespective of power position. This pattern corroborates previous findings that demonstrated merit-related effects on bargaining behavior and entitlements^23,31^. Nevertheless, the fact that positive and negative information exerted a similar, albeit opposite in direction, effect contradicts the well-documented asymmetric integration of information^49–51^. These results suggest that both powerful and powerless participants did not exploit contribution information to justify selfish behavior or to reduce dissonance between behavior and fairness considerations. A plausible reason for this pattern is that contribution information was relatively coarse (i.e., no information on the extent of contribution differences between the two players) and that differences in contribution were probably perceived minimal (i.e., task duration was the same for both players). Therefore, this type of information may not have allowed enough moral wiggle room for large deviations from the equality norm^19,30^. In fact, in all three experiments, powerful and powerless participants predominantly opted for an equal split or an allocation close to it. This pattern was also present in other studies^31,52^ and highlights the prominence of equality as a reference point of fairness^23^. In this respect, our experimental study constitutes a rather conservative test bed for power and merit effects. Unlike experimental settings, bargaining situations in the field can create strong entitlements based on merit and power hierarchies are usually perceived as stable and legitimate, both of which would likely reinforce the observed power-driven and merit-driven effects.

In Experiments 1 and 2, the relationship between the two power forms and fairness perceptions was fully (bargaining role) or partially (attractive outside options) mediated by bargaining behavior. On the other hand, unattractive outside options increased tolerance of self-disadvantageous allocations, at least for responders, but had no sizeable influence on fairness perceptions. Taken together, these findings suggest that people strive to minimize the dissonance between fairness considerations and selfish behavior by adopting self-serving interpretations of fairness, but they tolerate dissonance between self-undermining behavior and fairness considerations. One plausible explanation for this asymmetry is that decision power increases personal agency leading to a stronger need to reduce dissonance between selfish behavior and fairness considerations^8,9^. In this respect, the powerless are forced to tolerate self-disadvantageous allocations without being compelled to justify them, as they do not feel fully responsible for their actions. Personal agency could also explain why behavior fully mediated the bargaining role effect on fairness perceptions, whereas it only partially mediated the outside options effect. Bargaining roles clearly influence one’s agency in the distribution of resources, whereas outside options only have an indirect impact on one’s agency in resource allocation.

Similar to previous work^9,33^, Experiment 1 and 2 provide correlational evidence for the mediating role of bargaining behavior and thus do not preclude the possibility that fairness perceptions are formed ex-ante and influence bargaining behavior. Experiment 3 introduces a computer treatment, which eliminates alternative explanations (reverse causation or anchoring effects on bargaining behavior). Thus, Experiment 3 extends previous work by establishing the causal mediating role of bargaining behavior in the post-hoc power-driven adjustment of fairness perceptions.

The present work has important implications in the context of the rampant economic inequality worldwide. Many scholars attribute the absence of widespread public demand for wealth and income redistribution to the tendency of high-status and low-status individuals to legitimize inequalities by perceiving them as the outcome of meritocratic processes^5,53^. An alternative explanation posits that disadvantaged individuals recognize the unfairness of wealth distribution^54,55^, but the imbalance of political power between affluent and poor citizens^56^ instills a growing sense of powerlessness that results in political apathy^57^. Our findings are partly consistent with both explanations. On the one hand, procedural justice led powerless individuals to justify disadvantageous inequality. On the other hand, in the absence of power legitimacy, the extremely powerless accepted self-disadvantageous allocations but did not deem them fair. These results allude to an inherent instability of power-driven inequalities due to mutually inconsistent fairness perceptions of the powerful and powerless but also highlight the role of procedural justice in consolidating these inequalities.

A potential limitation of our study is the sample size which may not allow for a reliable identification of three-way interactions. We note, however, that the sample sizes were determined by a priori power analyses and all our preregistered hypotheses pertain to main effects or two-way interactions. The one-shot nature of our experiments and the exclusive focus on dyadic bargaining situations ignore the long-term and multi-party dynamics of power and thus limits the generalizability of the present results to more complex bargaining situations. Furthermore, our study focused on personal fairness perceptions and disregarded how power affects the perception of what others find fair (i.e., fairness norm). Given that fairness norms may substantially deviate from personal fairness perceptions and may exert different effects on behavior^58^, this distinction warrants further investigation. Lastly, the present work construes power as an opportunity for personal gain. However, recent evidence suggests that under certain conditions^59,60^ power can also be construed as responsibility for others which may in turn ameliorate selfish behavior and reduce the desire to obtain power^61^. Extending this work to our setting, future research should investigate how different forms of power are more or less amenable to the one or the other construal.

To conclude, the present work extends existing research by demonstrating an asymmetric power effect that contradicts the idea that power always bends the fairness perceptions of the weak and the strong in favor of the powerful. By operationalizing power in multiple ways, we identified an interesting synergistic effect of two common forms of power that should be further explored. Lastly, we demonstrated the role of procedural justice in the justification of inequalities and we demonstrated the causal mediating role of bargaining behavior in the power-driven formation of fairness perceptions.

## Supplementary Information

Supplementary Information.

## Data Availability

The datasets used and/or analysed during the current study available from the corresponding author on reasonable request.
