# Author Correction: Interplay between different forms of power and meritocratic considerations shapes fairness perceptions

**DOI:** 10.1038/s41598-023-29203-w

**Published:** 2023-02-02

**Authors:** Giannis Lois, Arno Riedl

**Affiliations:** 1 Department of Microeconomics and Public Economics, School of Business and Economics, Maastricht University, 6200 MD Maastricht, The Netherlands; 2 Maastricht University-Center of Neuroeconomics, Maastricht, The Netherlands; 3 CESifo, IZA, and Maastricht University, Maastricht, The Netherlands

Correction to: *Scientific Reports* 10.1038/s41598-022-15613-9, published online 06 July 2022

The Acknowledgements section in the original version of this Article was incomplete.

“We thank Katerina Petkanopoulou for her valuable comments.”

now reads:

“We thank Katerina Petkanopoulou for her valuable comments. Financial support was provided by the EU Horizon 2020 Marie Curie Individual Fellowship (Proposal Number: 895685) to G.L."

The original Article has been corrected.

