# Supplementary material for: Interplay between different forms of power and meritocratic considerations shapes fairness perceptions
Source: Sci Rep. 2022 Jul 6;12:11428. doi: 10.1038/s41598-022-15613-9 (PMC9259606; doi:10.1038/s41598-022-15613-9)
Supplement: Supplementary file 1 — Supplementary Information. [file 41598_2022_15613_MOESM1_ESM.docx]

**Title: Interplay between different forms of power and meritocratic considerations shapes fairness perceptions**

**Experiment 1**

**Participants**

Out of the 315 participants that were recruited, 247 were included in the analysis. The remaining participants were excluded because they failed to complete both sessions (see detailed Procedure below), did not answer correctly all comprehension questions, or failed to successfully complete the surplus production task.

**Procedure**

Participants took part in two separate sessions (four days apart). During the first session, participants performed a word-encoding task in which they have to encode as many words as possible into numbers within two minutes. The performance of proposers and responders -who were assigned to the effort condition- in the word-encoding task determined their assignment to the different DP conditions. More specifically, participants who performed better than their UG partner in the word-encoding task were assigned to the large-DP condition while those who performed worse than their UG partner were assigned to the small-DP condition. Matched pairs of proposers and responders who performed equally well were assigned to the equal-DP condition.

Following the belief elicitation phase of the main (second) session, participants were reminded of their stated fair allocation and were asked to provide arguments in favor of their response. Following the open-end question, participants also ranked a set of pre-selected arguments in terms of how well they explain their stated fair allocation. These data were not included in this study. At the end of the experiment, the ECUs that each player earned was exchanged to cash with a rate of 0.05£ for each ECU. On average, the experiment lasted 20 minutes and participants earned, on average, 2.50£.

**Experiment 2**

**Participants**

Out of the 426 participants that were recruited in this experiment, 372 were included in the final analysis. The remaining participants were excluded because they failed to answer correctly all comprehension questions.

**Procedure**

Following the belief elicitation phase, participants were reminded of their stated fair allocation and were asked to provide arguments in favor of their response. Following the open-end question, participants also ranked a set of pre-selected arguments in terms of how well they explain their stated fair allocation. These data were not included in this study. At the end of the experiment, the ECUs that each player earned was exchanged to cash with a rate of 0.05£ for each ECU. On average, the experiment lasted 20 minutes and participants earned, on average, 2.50£.

**Experiment 3**

**Participants**

Out of the 142 participants that were recruited in the computer condition, 123 were included in the final analysis. The remaining participants were excluded because they failed to answer correctly all comprehension questions or to successfully complete the surplus production task.

**Procedure**

Following the belief elicitation phase, participants were reminded of their stated fair allocation and were asked to provide arguments in favor of their response. Following the open-end question, participants also ranked a set of pre-selected arguments in terms of how well they explain their stated fair allocation. These data were not included in this study. At the end of the experiment, the ECUs that each player earned was exchanged to cash with a rate of 0.05£ for each ECU. On average, the experiment lasted 20 minutes and participants earned, on average, 2.50£.

**Tables**

**Table 1.** Number of participants per condition of Experiment 1.

|  | **Proposers** | | | **Responders** | | |
| --- | --- | --- | --- | --- | --- | --- |
|  | **Large DP** | **Equal DP** | **Small DP** | **Large DP** | **Equal DP** | **Small DP** |
| **Luck** | 20 | 21 | 21 | 21 | 20 | 21 |
| **Effort** | 21 | 21 | 18 | 21 | 23 | 19 |

**Table 2.** Means and standard deviations (in parentheses) of bargaining behavior and fairness perceptions across all conditions of Experiment 1.

|  |  | **Proposers** | | | **Responders** | | |
| --- | --- | --- | --- | --- | --- | --- | --- |
|  |  | **Large DP** | **Equal DP** | **Small DP** | **Large DP** | **Equal DP** | **Small DP** |
| **Bargaining behavior** | **Luck** | **60.6 (10.3)** | **51.1 (2.7)** | **48.0 (5.8)** | **49.6 (6.7)** | **45.2 (7.0)** | **32.6 (12.4)** |
|  | **Effort** | **60.7 (13.5)** | **53.1 (5.1)** | **48.6 (6.4)** | **54.0 (15.3)** | **46.0 (4.5)** | **33.3 (14.3)** |
| **Fairness perceptions** | **Luck** | **56.5 (10.4)** | **50.0 (2.7)** | **48.9 (5.0)** | **51.2 (6.9)** | **47.6 (6.4)** | **47.6 (7.0)** |
|  | **Effort** | **56.0 (9.8)** | **51.7 (4.6)** | **47.4 (5.2)** | **57.6 (8.6)** | **49.4 (1.8)** | **40.7 (13.6)** |

**Table 3.** Number of participants per condition of Experiment 2.

|  | **Proposers** | | | **Responders** | | |
| --- | --- | --- | --- | --- | --- | --- |
|  | **Large DP** | **Equal DP** | **Small DP** | **Large DP** | **Equal DP** | **Small DP** |
| **Advantageous** | 19 | 22 | 22 | 20 | 21 | 21 |
| **Ambiguous** | 22 | 22 | 23 | 19 | 21 | 19 |
| **Disadvantageous** | 20 | 19 | 21 | 20 | 20 | 21 |

**Table 4.** Means and standard deviations (in parentheses) of bargaining behavior and fairness perceptions across all conditions of Experiment 2.

|  |  | **Proposers** | | | **Responders** | | |
| --- | --- | --- | --- | --- | --- | --- | --- |
|  |  | **Large DP** | **Equal DP** | **Small DP** | **Large DP** | **Equal DP** | **Small DP** |
| **Bargaining behavior** | **Advantageous** | **59.6 (10.3)** | **53.6 (5.3)** | **50.5 (5.3)** | **55.8 (6.9)** | **47.2 (7.1)** | **39.0 (13.8)** |
|  | **Ambiguous** | **56.1 (14.0)** | **49.1 (3.0)** | **47.7 (7.3)** | **55.6 (12.4)** | **44.2 (8.0)** | **36.9 (13.2)** |
|  | **Disadvantageous** | **54.1 (12.9)** | **50.3 (13.1)** | **49.1 (5.0)** | **46.0 (10.1)** | **44.3 (6.3)** | **35.8 (10.5)** |
| **Fairness perceptions** | **Advantageous** | **58.8 (9.3)** | **52.0 (3.7)** | **51.6 (3.9)** | **53.9 (5.6)** | **50.2 (4.0)** | **49.0 (5.4)** |
|  | **Ambiguous** | **53.0 (9.2)** | **50.1 (2.0)** | **49.7 (6.9)** | **52.1 (6.7)** | **49.5 (3.5)** | **49.5 (1.6)** |
|  | **Disadvantageous** | **50.6 (7.2)** | **46.9 (4.4)** | **49.0 (4.9)** | **47.9 (7.1)** | **46.7 (4.3)** | **47.0 (11.2)** |

**Table 5.** Number of participants per condition of Experiment 3.

|  | **Proposers** | | | **Responders** | | |
| --- | --- | --- | --- | --- | --- | --- |
|  | **Large DP** | **Equal DP** | **Small DP** | **Large DP** | **Equal DP** | **Small DP** |
| **Human** | 20 | 21 | 21 | 21 | 20 | 21 |
| **Computer** | 20 | 20 | 21 | 20 | 21 | 21 |

**Table 6.** Means and standard deviations (in parentheses) of fairness perceptions across all conditions of Experiment 3.

|  | **Proposers** | | | **Responders** | | |
| --- | --- | --- | --- | --- | --- | --- |
|  | **Large DP** | **Equal DP** | **Small DP** | **Large DP** | **Equal DP** | **Small DP** |
| **Human** | **56.5 (10.4)** | **50.0 (2.7)** | **48.9 (5.0)** | **51.2 (6.9)** | **47.6 (6.4)** | **47.6 (7.0)** |
| **Computer** | **51.8 (4.9)** | **50.2 (1.1)** | **50.2 (3.9)** | **49.6 (1.3)** | **50.0 (1.7)** | **50.0 (0.0)** |
